# Phylogenomic analysis of *Phytophthora cinnamomi* collected from avocado in the United States reveals links to the two A2 panglobal clonal lineages

**DOI:** 10.3389/fpls.2026.1870818

**Published:** 2026-07-21

**Authors:** Benjamin K. Hoyt, Aidan C. Shands, Jonathan H. Crane, Monica Navia-Urrutia, Romina Gazis, Liliana M. Cano, Achyut Adhikari, Miaoying Tian, John Jifon, Ricardo Goenaga, Luz M. Serrato-Diaz, Niklaus J. Grünwald, Patricia Manosalva

**Affiliations:** 1 Department of Microbiology and Plant Pathology, University of California, Riverside, Riverside, CA, United States; 2 Tropical Research and Education Center, Institute of Food and Agricultural Sciences, University of Florida, Homestead, FL, United States; 3 Department of Plant Pathology, Indian Research and Education Center, Institute of Food and Agricultural Sciences, University of Florida, Fort Pierce, FL, United States; 4 Department of Plant and Environmental Protection Sciences, University of Hawaii at Mānoa, Honolulu, HI, United States; 5 Department of Horticultural Sciences, Texas A&M AgriLife Research, Weslaco, TX, United States; 6 USDA-Agricultural Research Service (ARS), Tropical Agriculture Research Station, Mayaguez, Puerto Rico; 7 Horticultural Crops Disease and Pest Management Research Unit, USDA-Agricultural Research Service (ARS), Corvallis, OR, United States

**Keywords:** genetics, multilocus genotypes, oomycete, ploidy, population structure

## Abstract

*Phytophthora cinnamomi*, the causal agent of phytophthora root rot (PRR), is one of the most destructive pathogens affecting avocados worldwide. Recent global population analyses of *P. cinnamomi* identified two panglobal clonal lineages named PcG1-A2 and PcG2-A2, both of A2 mating type. In California avocado orchards, *P. cinnamomi* populations were previously classified into two clades, A2 clade I and A2 clade II, with the latter likely introduced from Mexico. In this study, we conducted the genetic characterization of 125 isolates collected from avocado-producing regions of the United States and investigated their genetic relationships with previous populations from California and Mexico and with the two panglobal A2 clonal lineages using whole-genome resequencing. All isolates were estimated to be triploid based on nQuire. Phylogenetic reconstruction and fastSTRUCTURE analyses based on single nucleotide polymorphisms showed that the PcG1-A2 reference isolate clustered with the previously described California A2 clade I and with most U.S. isolates. In contrast, the PcG2-A2 reference isolate clustered with the California A2 clade II and with most Mexican isolates, supporting a Mexican origin for this lineage. Discriminant analysis of principal components, principal coordinate analysis, and minimum spanning networks further resolved two distinct groups of A2 isolates, reinforcing the presence of two clonal lineages in avocado orchards across the United States. Isolates were assigned to multilocus genotypes (MLGs), including one widespread MLG containing isolates from all sampled avocado-growing regions. Mexico exhibited the highest genetic diversity, although unique MLGs were detected at each location. These results clarify the genetic relationships among *P. cinnamomi* populations in avocado orchards across the United States and link the previously described California clades to the two panglobal lineages currently recognized. Our findings support a hypothesis of pathogen movement among avocado-producing regions and reveal localized subpopulations that may pose risks to the sustainability of the avocado industry. Continued monitoring of *P. cinnamomi* populations will be essential for developing effective PRR management strategies to increase avocado production and enhance the national industry’s profitability, competitivity, and marketability.

## 1 Introduction

Phytophthora root rot (PRR) of avocado caused by *Phytophthora cinnamomi*, is one of the most significant production challenges for avocado worldwide. The global avocado market has increased rapidly over the last decade, and global production is projected to reach roughly 12 million metric tons by 2030 (Huang et al., 2023). Mexico has remained the largest avocado producer in the world over the past several decades (FAO, 2025), although avocado production has expanded to other countries in Latin America, Europe, and Asia (Magaña et al., 2025). Avocado consumption in the United States has also increased substantially in recent decades, outpacing domestic production, which is limited to regions with warmer climates such as California, Florida, Hawaii, Texas, and Puerto Rico (Hammami et al., 2024). In Mexico, *P. cinnamomi* has been largely responsible for avocado tree mortality, particularly in Michoacán, where approximately 70% of national avocado production occurs (Mondragón-Flores et al., 2022). Similarly, 60-75% of avocado growers in California, the top avocado-producing state in the United States, are affected by this oomycete pathogen (Coffey, 1987; Ploetz, 2013).

*P. cinnamomi* is a destructive soil-borne pathogen that infects over 5,000 plant species, including forest trees and crops such as avocado (Hardham and Blackman, 2018). This highly invasive pathogen causes significant economic losses to the agricultural industry, with crops such as avocado, chestnut, macadamia, peach, and pineapple among those most severely affected (Hardham, 2005). *P. cinnamomi* is a heterothallic species that requires two mating types (A1 and A2) for sexual reproduction; however, it primarily reproduces asexually through the production of non-caducous sporangia that release motile zoospores, which serve as the primary infective propagules (Hardham and Blackman, 2018). The pathogen also produces chlamydospores, thick-walled survival structures that enables persistence under unfavorable environmental conditions (McCarren et al., 2005). Infection primarily occurs in feeder roots, leading to root system decline, reduced fruit yield, and overall deterioration of tree health, that can ultimately result in tree mortality if not properly managed (Ploetz and Parrado, 1987). Due to its broad host range, persistence in soil, and capacity to spread through water, soil movement, and infected nursery material, *P. cinnamomi* has become widely distributed in avocado-producing regions.

Current PRR management strategies rely on an integrated approach that includes the use of registered Oomycota-targeting fungicides, tolerant or resistant rootstock cultivars, and improved cultural practices (Dann and Gazis, 2024). Common chemical treatments include phosphonate-based products, mefenoxam, and oxathiapiprolin (Adaskaveg et al., 2024; Crane et al., 2024). In addition to chemical control, cultural practices such as optimized irrigation management, mulching, gypsum applications, planting on raised berms, and routine disease diagnostics are widely recommended to reduce disease pressure and improve orchard health (Dreistadt, 2007). Together, these strategies aim to limit pathogen spread, reduce infection rates, and enhance tree resilience to PRR.

Clonal populations of the A2 mating type of *P. cinnamomi* are frequently reported to exhibit increased virulence and have been strongly associated with major avocado PRR epidemics worldwide (Dobrowolski et al., 2003; Jung et al., 2013; Engelbrecht et al., 2022; Shands et al., 2025). Studies from long-established avocado-growing regions have reported *P. cinnamomi* isolates with increased virulence toward commonly planted rootstock cultivars (Belisle et al., 2019a; Hoyt et al., 2026a; Sánchez-González et al., 2019). In addition, reduced sensitivity to potassium phosphite has been documented in several *P. cinnamomi* populations (Adaskaveg et al., 2024; Andronis et al., 2024; Calle-Henao et al., 2020; Dobrowolski et al., 2008; Shands et al., 2025; Hoyt et al., 2026b). Notably, populations exhibiting increased virulence or reduced sensitivity to potassium phosphite belong to the A2 mating type (Dobrowolski et al., 2008; Shands et al., 2025). These clonal A2 populations are capable of rapid dispersal through infected nursery material, contaminated soil, and water movement within orchards, facilitating the establishment of widespread outbreaks.

Recent advances in whole genome sequencing have enabled high-resolution phylogenomic analyses that provide deeper insights into the evolutionary history, population structure, and global dispersal of *P. cinnamomi* (Engelbrecht et al., 2022; Shakya et al., 2021; Shands et al., 2025). The geographic origin of this pathogen has long been debated. *P. cinnamomi* was first described from Sumatra, where it was associated with striped cankers on cinnamon trees (Rands, 1922). Early hypotheses proposed that the pathogen originated in Southeast Asia based on its association with native vegetation (Crandall and Gravatt, 1967; Ko et al., 1978). Other studies suggested that the center of origin may lie in the Australasian region, particularly Papua New Guinea and parts of Indonesia, where the pathogen occurs in diverse ecosystems (Shepherd, 1975). The center of origin of *P. cinnamomi* has been recently proposed to be near Taiwan and Vietnam, based on the high genetic diversity and the presence of both A1 and A2 mating types at approximately equal frequencies (Shakya et al., 2021). In addition, Shakya et al. (2021), also proposed a model in which Australia, South Africa, and Papua New Guinea *P. cinnamomi* populations with reduced genetic diversity and sexual reproduction served as stepping-stone for the migrations of clonal populations of this pathogen to other regions including the Americas. In the same study, two widely distributed panglobal clonal lineages of the A2 mating type, designated PcG1-A2 and PcG2-A2, were identified across multiple continents. This is consistent with earlier regional studies that also reported the presence of two A2 clonal pathogen populations.

Dobrowolski et al. (2003) identified two major A2 clonal lineages in Australia, referred to as A2 type 1 and A2 type 2, which were associated with disease outbreaks in native *Banksia* woodlands. Similar patterns have been observed in avocado production systems. Pagliaccia et al. (2013), revealed *P. cinnamomi* isolates from California avocado orchards clustered into two distinct A2 clonal lineages, designated A2 clade I and A2 clade II. A2 clade I represented a historic pathogen population primarily associated with avocado-growing regions in Northern California, whereas A2 clade II was a more recently introduced population detected in Southern California counties including Riverside and San Diego. Subsequent genomic analyses by Shands et al. (2025) provided evidence supporting the Mexican origin for the A2 clade II population present in Southern California. However, it remains unclear whether these two A2 clades associated with avocado PRR represent the two previously described panglobal A2 clonal lineages, independent introduction events, or distinct evolutionary groups. Furthermore, population structure and genetic relationships of *P. cinnamomi* in other U.S. avocado-producing regions have not been comprehensively assessed.

To address these knowledge gaps, we genetically characterized populations of *P. cinnamomi* collected from avocado orchards in California, Florida, Hawaii, Texas, and Puerto Rico, and compared them with isolates from Mexico and two reference isolates representing the panglobal A2 clonal lineages, PcG1-A2 and PcG2-A2. We evaluated the population structure, genetic differentiation, and dominant ploidy of *P. cinnamomi* across these regions to determine how pathogen populations from California and Mexico relate to those present in other U.S. avocado-growing areas. Understanding the genetic relationships among *P. cinnamomi* populations associated with avocado production is essential for improving disease management strategies. Monitoring pathogen population diversity will help guide the development and deployment of avocado rootstocks with durable resistance to genetically diverse pathogen populations. Furthermore, the genomic resources generated in this study will facilitate the development of diagnostic markers for the detection of important *P. cinnamomi* genotypes, supporting biosurveillance efforts aimed at protecting the long-term sustainability of the U.S. avocado industry.

## 2 Materials and methods

### 2.1 Pathogen isolates, genomic DNA extraction, and sequencing

The majority of the *P. cinnamomi* isolates used in this study were obtained from the Manosalva Laboratory collection at the University California, Riverside. Isolates were stored long-term on 10% clarified V8 agar plugs (7-mm diameter) suspended in 900 μl of sterile water in a 2-ml screw-cap tube, which were stored at 22 °C in darkness. A total of 127 isolates were included in this study, comprising the 125 isolates phenotypically characterized by Hoyt et al. (2026b) and two *P. cinnamomi* isolates representative of the PcG1-A2 and PcG2-A2 panglobal clonal lineages defined by Shakya et al. (2021) (**Supplementary Table 1**). All *P. cinnamomi* isolates were propagated in 10 ml of liquid Plich media (Van der Lee et al., 1997) amended with 50 µg/ml penicillin G (Gold Biotechnology, St. Louis, MO, U.S.A.) and grown for 4 to 6 days at 22 °C in complete darkness. The mycelium was rinsed, collected, and stored following the methods described in Shands et al. (2025). Genomic DNA was extracted using the DNeasy Plant Mini Kit (Qiagen, Germantown, MD, U.S.A.) following the manufacturer’s instructions, with modifications outlined in Shands et al. (2025). Library preparation (350-bp insert) and whole-genome sequencing at 30× coverage (150-bp paired-end reads) were performed by Novogene Corporation (Sacramento, CA, U.S.A.) using the Illumina NovaSeq platform.

### 2.2 Read mapping and quality filtering

Raw reads corresponding to the 127 isolates sequenced in this study were provided by Novogene with adapters removed and partially filtered. Additional filtering was performed using fastq-mcf v.1.05 (Aronesty, 2011) with a quality threshold of -q 20. Filtered reads were then mapped to the *P. cinnamomi* isolate Pc2113 reference genome (140.7 Mb) (Shands et al., 2024) using BWA-mem v.0.7.17 (Li, 2013). Mapping percentages were calculated, and the resulting BAM files were sorted and marked for duplicate reads using SAMtools (Li et al., 2009), following the procedures described in Shands et al. (2025).

### 2.3 Variant calling, filtering, and annotation

These analyses were conducted by combining the sequencing data corresponding to the 127 isolates from this study and the whole-genome Illumina sequencing data for the 136 P*. cinnamomi* isolates analyzed by Shands et al. (2025) downloaded from the NCBI Bioproject PRJNA1022429. Single-nucleotide polymorphisms (SNPs) were identified for each isolate using the Genome Analysis Toolkit (GATK; version 4.2.5.0; DePristo et al., 2011) in a two-step variant calling workflow described by Shands et al. (2025). Variants were initially called using the HaplotypeCaller module with the -ERC GVCF option and a ploidy level of two. Resulting GVCF files were merged using the MergeVcfs function in Picard Tools (v2.26.11), and joint genotyping was performed using the GenotypeGVCFs function in GATK. The resulting VCF files were then merged again using Picard MergeVcfs to generate a single combined VCF file. Filtering was done in two steps on the combined VCF file, first, by using GATK SelectVariants, retaining only biallelic SNPs, removing unused alternate alleles, and excluding non-variant sites. The second filtering step was conducted using VCFtools (v0.1.16–18) in several steps as described in Shands et al. (2025). The filtering with VCFtools removed SNPs with read depth <4 (--minDP 4), retained SNPs with quality scores >20 (--minQ 20), required no missing data (--max-missing 1), excluded singleton positions (--exclude-positions), and removed SNPs located within annotated repetitive regions (--exclude-bed) using a BED file generated from the repeat annotations of the *P. cinnamomi* isolate Pc2113 reference genome (Shands et al., 2024) with BEDOPS v.2.4.40 (Neph et al., 2012). Filtering the combined VCF file resulted in 200,345 SNPs that were used for all the analyses in this study.

### 2.4 Ploidy estimation

Ploidy estimation was performed using nQuire v.2018-04-05-a990a88 (Weiß et al., 2018) following the parameters described by Shands et al. (2025). BAM files generated during variant calling were used as input, and mean sequencing coverage was calculated with Mosdepth v.0.3.3 (Pedersen and Quinlan, 2018). Isolate Pc2113, previously described as a triploid (Shands et al., 2024) was included as a control.

### 2.5 Population genomic analyses

Population structure among *P. cinnamomi* isolates was investigated using multiple complementary approaches using R v.4.3.2 (R Core Team, 2023). Principal component analysis (PCA) was performed using *glPca* function in the R package *adegenet* v.2.1.7 (Jombart, 2008; Jombart and Ahmed, 2011). Population clustering was further examined using discriminant analysis of principal components (DAPC), implemented in *adegenet*. An unrooted phylogenetic tree was constructed using the unweighted pair group method with arithmetic mean (UPGMA) implemented in *poppr* v.2.9.3 (Kamvar et al., 2014), based on bitwise genetic distances with 1,000 bootstrap replicates. In addition, a minimum spanning network (MSN) was generated using *poppr* with bitwise distance to visualize relationships among isolates. Population structure was inferred using fastSTRUCTURE (Raj et al., 2014) with the simple prior testing values of genetic clusters (K) ranging from 1 to 10. The optimal K value was estimated using the *ChooseK* Python script included with fastSTRUCTURE. Based on the *Choose*K results, fastSTRUCTURE was rerun using the log prior to detect subtle population structure, testing K values ranging from 2 to 5. A second ChooseK analysis was then conducted to determine the final K value used for interpretation. Population genetic differentiation was evaluated by calculating fixation index (*F_ST_*) analogs, *G_ST_* (Nei, 1972; Nei, 1973) and *G′_ST_* (Hedrick, 2005), using the *mmod* package (Winter, 2012). Prior to clone correction, multilocus genotype (MLG) filtering was performed using a genetic distance threshold of 0.01 with the mlg.filter function, followed by clone correction using the clonecorrect function in *poppr*. Except for fastSTRUCTURE analyses, all population genomic analyses were conducted using both clone-corrected and non-clone-corrected datasets.

## 3 Results

### 3.1 Number of variants and ploidy levels of *P. cinnamomi* isolates from U.S. avocado-growing regions

A total of 127 isolates were sequenced in this study, including 125 P*. cinnamomi* isolates originated from California (n = 38), Florida (n = 25), Hawaii (n = 12), Texas (n = 25), and Puerto Rico (n = 25) (Hoyt et al., 2026b), as well as two isolates from Oregon (R024 and R001) representing the two A2 panglobal clonal lineages, PcG1-A2 and PcG2-A2, respectively. Whole-genome sequencing generated an average of 4.01 ± 0.96 Gb of filtered data per isolate, with an average mapping rate of 97.1 ± 1.9% (**Supplementary Table 1**). Variant calling of 263 isolates used in this study initially identified approximately 3.9 million raw SNPs, of which 200,345 high-quality SNPs passed filtering and were retained for all downstream analyses. All 127 newly sequenced isolates were estimated to be triploid based on the ratio of minor to major allele frequencies inferred from read counts, exhibiting a ∆log average value 48-fold lower than those of the other models tested (**Supplementary Table 2**).

### 3.2 Population structure and phylogenetic reconstruction of *P. cinnamomi* from avocado-growing regions in the United States

**Figure 1:**
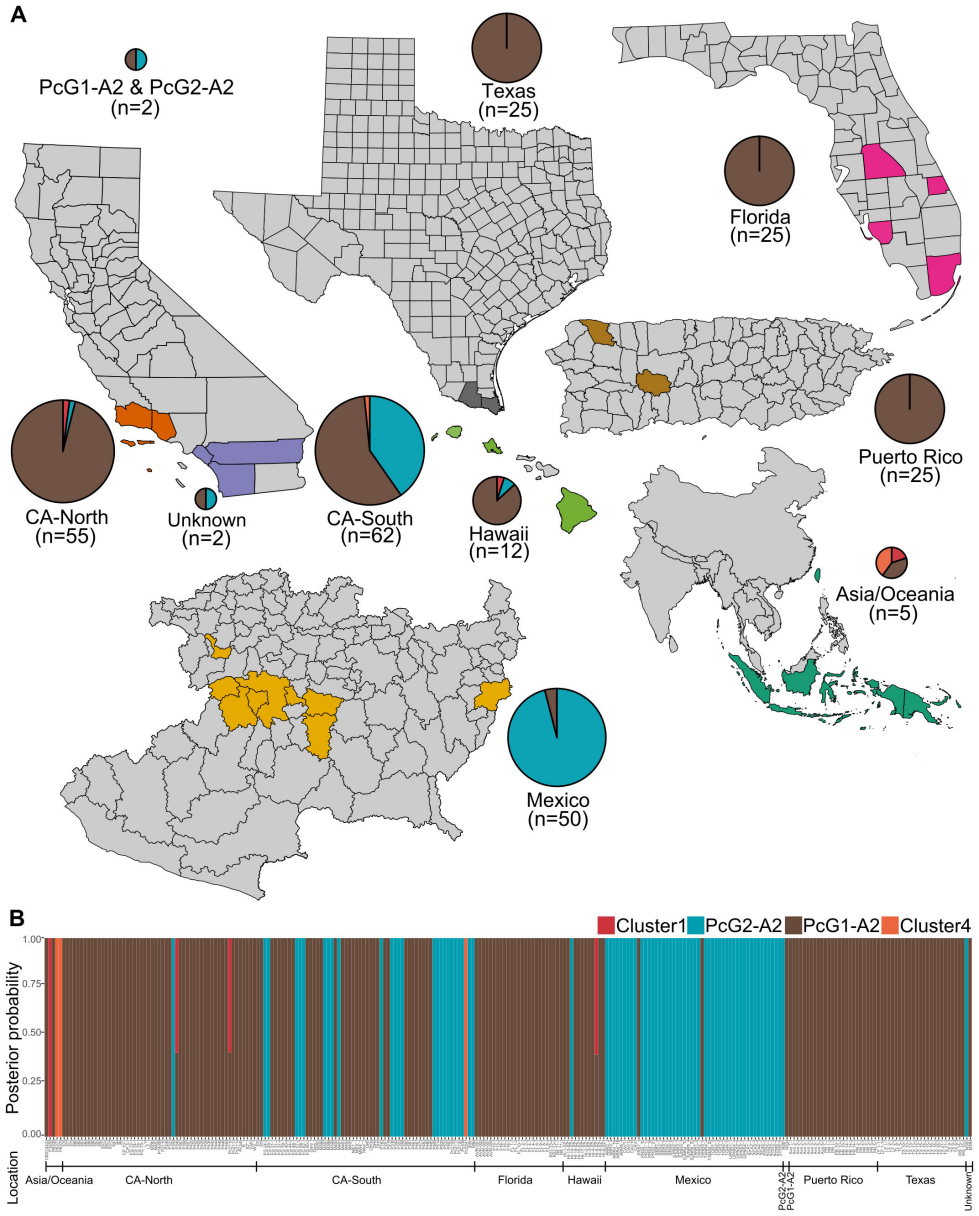
Population structure of *P. cinnamomi* collected from avocado orchards in the U.S.A. and Mexico. **(A)** Geographic location from which the *P. cinnamomi* isolates were collected. Pie charts represent the average membership fractions of each location’s population based on an ADMIXTURE plot of K = 4, scaled to the number of samples. The PcG1-A2 and PcG2-A2 panglobal lineages are shown in brown and blue, respectively. Regions are highlighted in colors assigned to different sample locations (Asia/Oceania = teal, CA-North = orange, CA-South = blue, Florida = pink, Hawaii = green, Mexico = yellow, Puerto Rico = brown, and Texas = gray). Maps were downloaded from mapchart.net. **(B)** ADMIXTURE plot of *P. cinnamomi* isolates for K = 4 populations generated by fastSTRUCTURE. Genetic clusters are indicated by colors. Each bar represents the isolate’s estimated membership fractions to each genetic cluster. Membership probabilities from 0 to 1 are indicated on the Y-axis, while geographical regions are shown on the X-axis.

A total of 263 P*. cinnamomi* isolates were analyzed using whole-genome sequencing data. This dataset included 125 isolates collected from avocado orchards across major U.S. production regions (Hoyt et al., 2026b) (**Supplementary Table 1**), 136 isolates representing diverse locations, hosts, mating types, and collection years (Shands et al., 2025), as well as reference isolates corresponding to the A2 panglobal lineages (PcG1-A2 and PcG2-A2) (**Figure 1A**). Genetic assignment of *P. cinnamomi* isolates to the panglobal A2 clonal lineages described by Shakya et al. (2021) was assessed using multiple analytical approaches. Population structure analysis using fastSTRUCTURE identified optimal K values ranging from 3 to 5 with similar clustering patterns (**Supplementary Figure 1**); however, K = 4 was selected as capturing the general population structure best. The majority of isolates belonged to two genetic clusters corresponding to the PcG1-A2 panglobal lineage (brown) and the PcG2-A2 panglobal lineage (blue) (**Figure 1**). A third genetic cluster (red) consisted of a single A1 isolate from Papua New Guinea (PNG; P3658), while a fourth genetic cluster (orange) comprised two isolates from Taiwan, one A1 (P6379) and one A2 (P6377), together with an A1 isolate from Southern California (Pc5241) (**Figure 1B**). Most isolates from Northern California, Florida, Hawaii, Texas, and Puerto Rico, as well as A2 isolates from Papua New Guinea (P6339) and Indonesia (CBS 144.22), clustered with the PcG1-A2 lineage. In contrast, most isolates from Mexico, approximately 40% of isolates from the Southern California region (CA-South), one isolate from the Northern California (CA-North) (P344), and one Hawaiian isolate (HI-14-SZ1) clustered with the PcG2-A2 lineage. Notably, two A2 isolates from CA-North (Pc2110 and P349) and one Hawaiian isolate (HI-5-SZ1) showed mixed ancestry, sharing ~40% ancestry with the PcG1-A2 cluster and ~60% with the genetic cluster containing the A1 PNG isolate (P3658) (**Figure 1**; **Supplementary Figure 1**).

**Figure 2:**
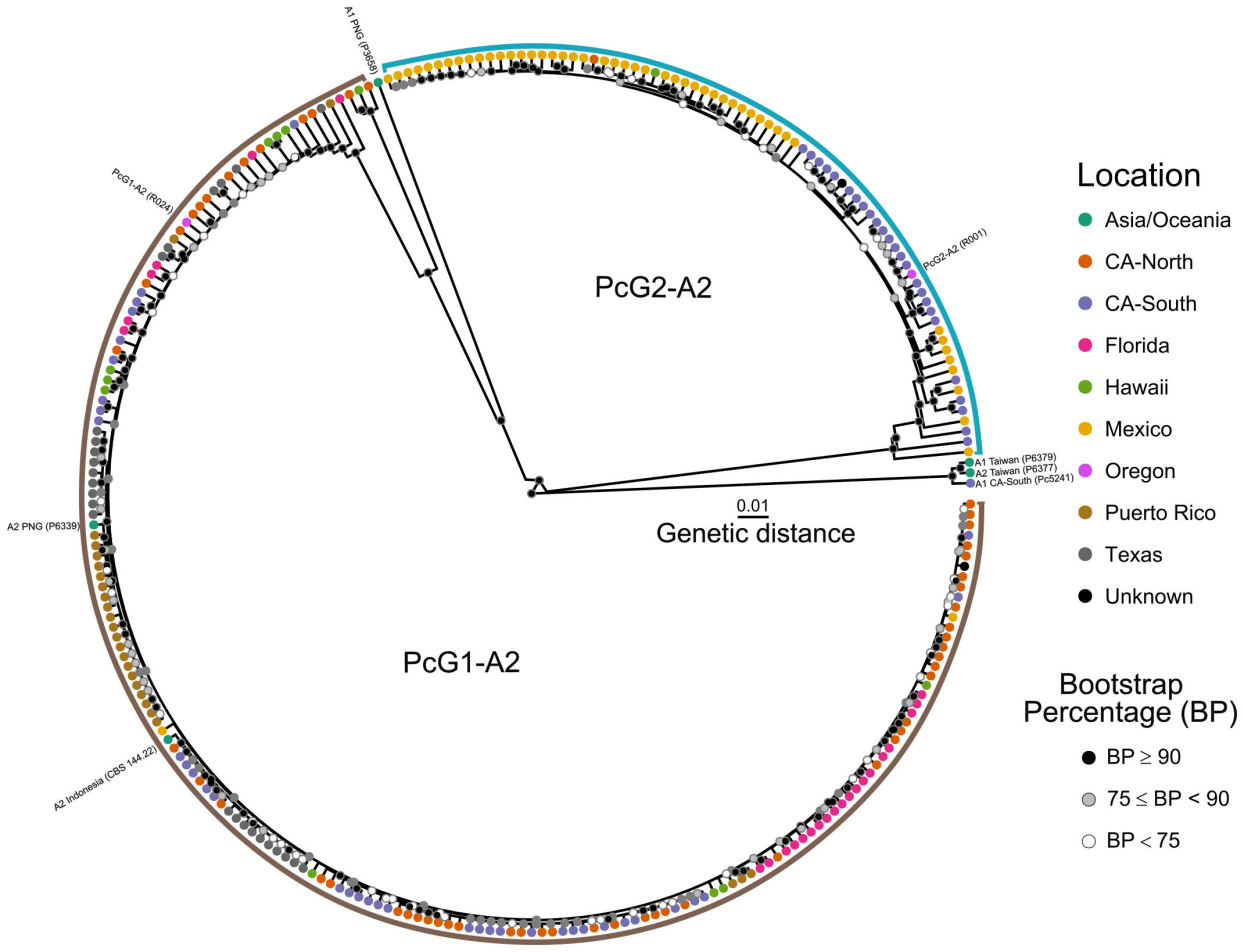
Phylogeny of *P. cinnamomi* populations from avocado orchards in the United States and Mexico displayed as an unrooted unweighted pair group method with arithmetic mean tree with 1,000 bootstrap replicates. Each sample in the tree node is color coded by its geographical location. Bootstrapping percentage and genetic distance are also indicated. The two *P. cinnamomi* A2 panglobal clonal lineages are outlined in brown (PcG1-A2) and blue (PcG2-A2). Reference A1 and A2 isolates used in this study are also indicated with the isolate ID in parentheses.

Phylogenetic reconstruction using the unweighted pair group method with arithmetic mean (UPGMA) supported the presence of the two A2 panglobal clonal lineages (**Figure 2**). The PcG1-A2 lineage included isolates from all major avocado-growing regions of the United States (CA-North, CA-South, Florida, Hawaii, Texas, and Puerto Rico), two isolates from Mexico, the A2 isolates from PNG (P6339) and Indonesia (CBS 144.22), and the reference isolate R024. The PcG2-A2 lineage was mostly comprised of most Mexican isolates and several isolates from CA-South, as well as one isolate from CA-North (P344), one Hawaiian isolate (HI-14-SZ1), and the reference isolate R001 (**Figure 2**). The three admixed isolates clustered near the PcG1-A2 clade. In contrast, the Taiwanese isolates (A1 P6379 and A2 P6377), together with the A1 isolate from Southern California, formed a separate cluster and were the most genetically distant from the two A2 panglobal clonal lineages (**Figure 2**).

### 3.3 Minimal genetic differentiation among *P. cinnamomi* populations from the U.S. and Mexico

To further assess population structure, clone correction was applied to collapse isolates into representative multilocus genotypes (MLGs). The 263 isolates were assigned to 61 distinct MLGs based on SNP similarity. Several MLGs were region-specific (n = >2 from the same region), shared among regions, and unique (single isolate). The U.S. population was assigned to 41 MLGs. Mexico contained the highest number of unique MLGs (13), followed by CA-North (12), CA-South (9), Florida (2), Texas (2), Hawaii (2), and Puerto Rico (2). Notably, 141 isolates representing all avocado production regions, along with two A2 isolates from Papua New Guinea and one isolate from an unknown location in California, were collapsed in a single MLG (MLG215) (**Supplementary Table 3**). Despite the high clonality observed, genetic differentiation among populations was estimated using Nei’s *G_ST_* and Hedrick’s *G′_ST_*, where values closer to 0 indicate low differentiation and values closer to 1 indicate high differentiation (**Table 1**). Overall, pathogen populations showed limited genetic differentiation (*G_ST_*= -0.01 to 0.216; *G′_ST_* = -0.02 to 0.462). With the exception of CA-South, the highest differentiation was observed between populations from Mexico and Puerto Rico (*G_ST_* = 0.216, *G′_ST_* = 0.462), Texas (*G_ST_* = 0.216, *G′_ST_* = 0.461), Florida (*G_ST_* = 0.215, *G′_ST_* = 0.46), CA-North (*G_ST_* = 0.205, *G′_ST_* = 0.462), and Hawaii (*G_ST_* = 0.164, *G′_ST_* = 0.376). These results indicate that Mexican isolates were more closely related to those from CA-South (*G_ST_* = 0.071, *G′_ST_* = 0.181) than to isolates from other U.S. regions (**Table 1**). Clone correction did not substantially change patterns of genetic diversity, population relationships, or the magnitude of differentiation among *P. cinnamomi* populations (**Table 1**).

**Table 1:** Population differentiation based on Nei’s *G_ST_* and Hedrick’s *G′_ST_*^a^.

Parameter	Asia/Oceania	CA-North	CA-South	Florida	Hawaii	Mexico	Puerto Rico
Nei’s *G_ST_*							
CA-North	0.041 (0.145)						
CA-South	0.018 (0.008)	0.035 (0.054)					
Florida	0.044 (0.16)	-0.006 (-0.03)	0.038 (0.07)				
Hawaii	0.021 (-0.01)	-0.009 (-0.24)	0.013 (0.017)	-0.01 (-0.03)			
Mexico	0.116 (0.09)	0.205 (0.16)	0.071 (0.013)	0.215 (0.18)	0.164 (0.11)		
Puerto Rico	0.044 (-0.002)	-0.004 (-0.04)	0.039 (0.05)	-0.008 (-0.06)	-0.008 (-0.05)	0.216 (0.17)	
Texas	0.043 (0.016)	-0.005 (-0.03)	0.038 (0.07)	-0.008 (-0.05)	-0.009 (-0.03)	0.215 (0.18)	-0.008 (-0.06)
Hedrick’s *G′_ST_*^a^							
CA-North	0.108 (0.041)						
CA-South	0.051 (0.023)	0.091 (0.143)					
Florida	0.115 (0.043)	-0.015 (-0.071)	0.01 (0.169)				
Hawaii	0.058 (-0.031)	-0.024 (-0.068)	0.04 (0.049)	-0.026 (-0.082)			
Mexico	0.289 (0.227)	0.445 (0.366)	0.181 (0.036)	0.46 (0.411)	0.376 (0.265)		
Puerto Rico	0.116 (-0.005)	-0.011 (-0.115)	0.102 (0.131)	-0.02 (-0.179)	-0.022 (-0.131)	0.462 (0.386)	
Texas	0.114 (0.044)	-0.013 (-0.070)	0.01 (0.170)	-0.022 (-0.130)	-0.024 (-0.082)	0.461 (0.412)	-0.02 (-0.183)

^a^ Values in parentheses were calculated based on clone-corrected data.

### 3.4 Multivariate analyses and minimum spanning networks confirmed linkage of U.S. and Mexican *P. cinnamomi* populations to A2 panglobal clonal lineages

**Figure 3:**
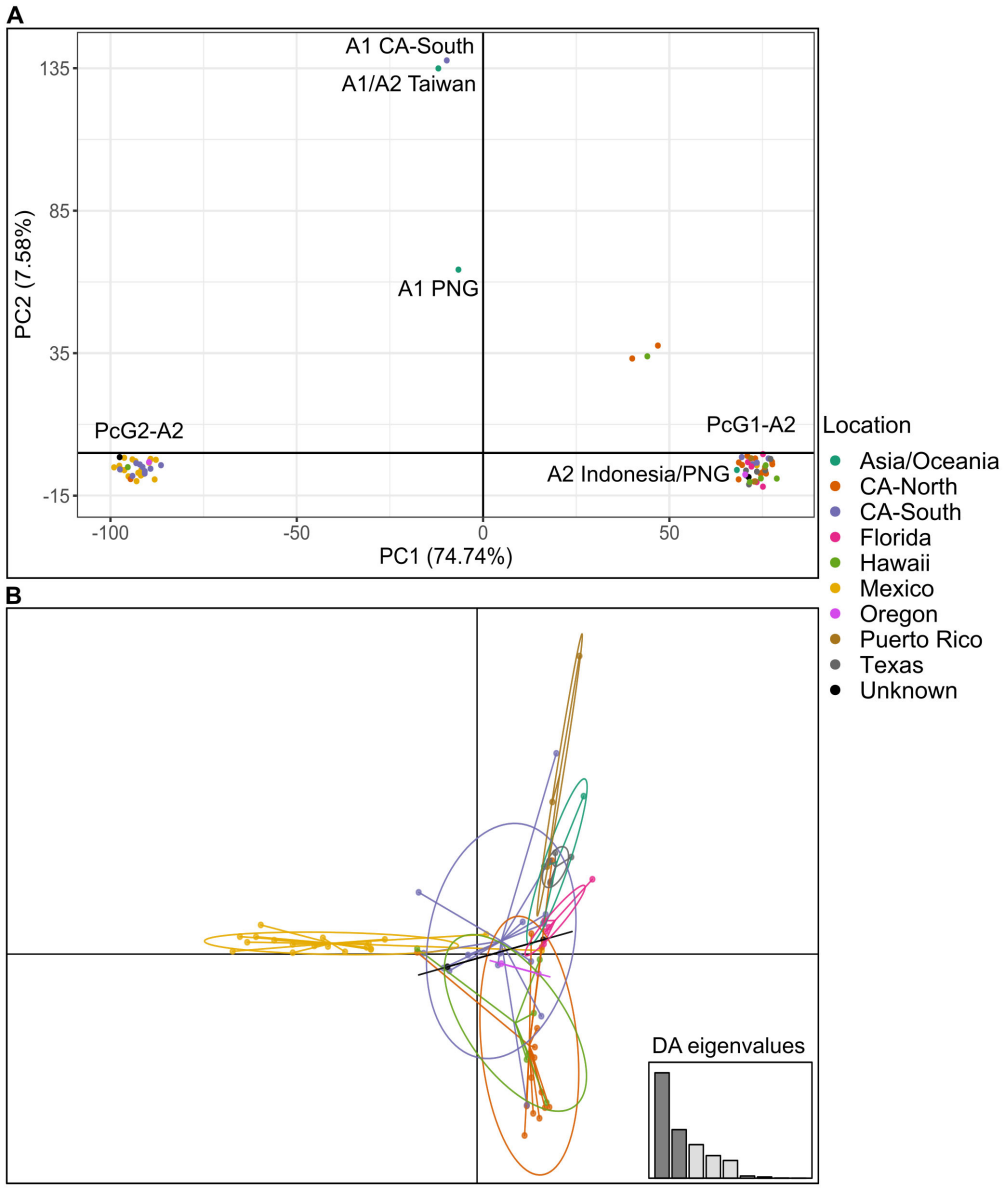
Multivariate analyses of the *P. cinnamomi* populations from the U.S.A. and Mexico after clone correction. **(A)** Principal component analysis (PCA) plot showing two principal components, in which PC1 reflects 74.74%, and PC2 reflects 7.58% of the total variation. *Phytophthora cinnamomi* reference isolates corresponding to the two A2 panglobal clonal lineages (PcG1-A2 and PcG2-A2), several A1 and A2 isolates from Taiwan, Papua New Guinea (PNG), and Indonesia, as well as an A1 isolate from CA-South collected from Camelia are indicated. **(B)** Discriminant analysis of principal components (DAPC) plot indicating the different PC groups, where the points represent the individuals and the ellipses represent the geographical locations. DA eigenvalues distribution is also displayed. Points in each plot reflect the individual by geographical location: Asia/Oceania = teal, CA-North = orange, CA-South = blue, Florida = pink, Hawaii = green, Mexico = yellow, Oregon = purple, Puerto Rico = brown, Texas = gray, and Unknown region from California = black.

**Figure 4:**
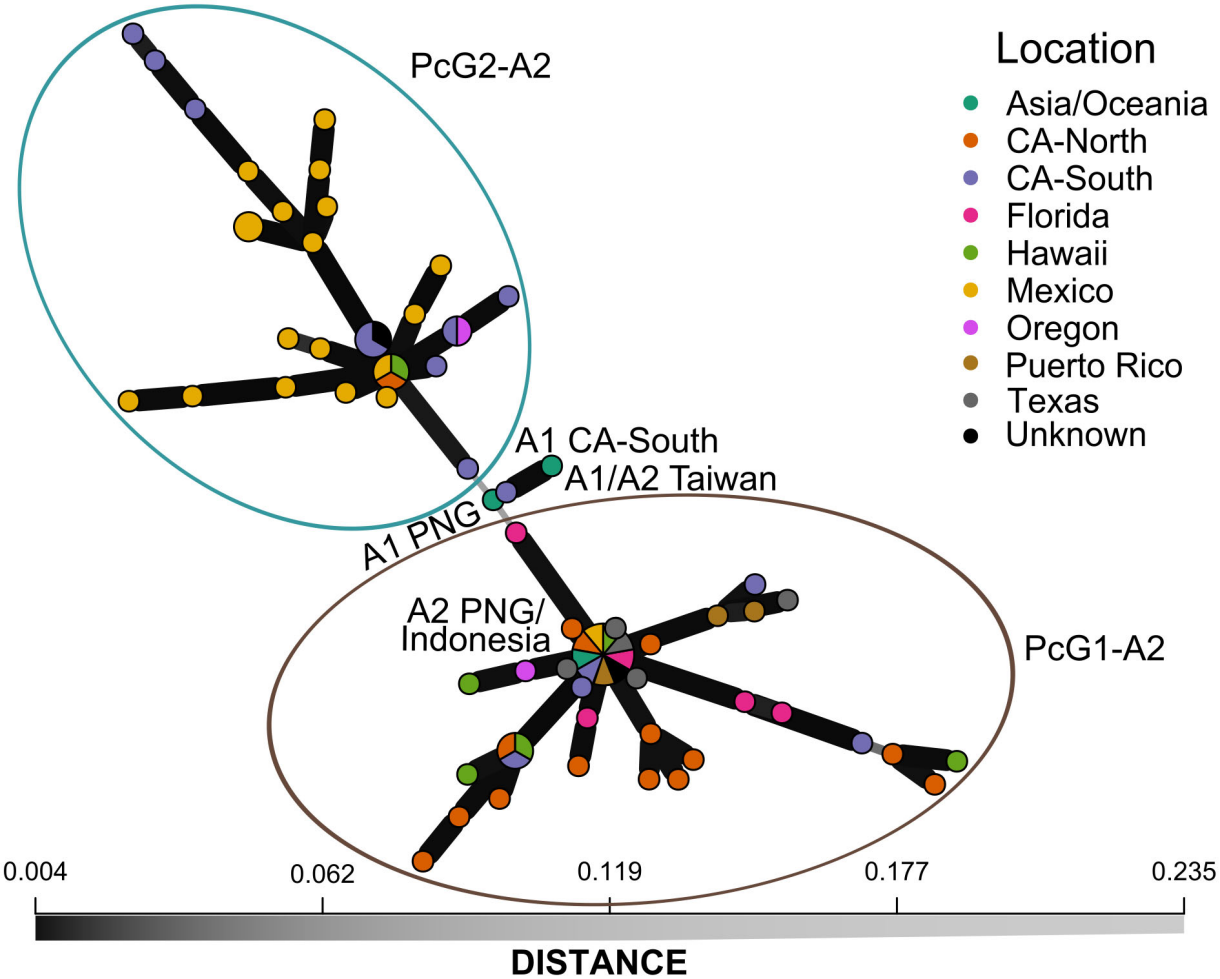
Minimum spanning network (MSN) analysis of multilocus genotypes (MLGs) showing the genetic relationships across samples from different locations and collection years. The MSN plot was generated using pairwise distances between each sample, where edge thickness represents the genetic distance. *Phytophthora cinnamomi* A2 panglobal clonal lineages are displayed as brown (PcG1-A2) and blue (PcG2-A2) ellipses. Nodes containing reference isolates from Asia and Oceania are also indicated. Nodes in each plot represent a single MLG, with node size proportional to the number of representative genotypes. Pie chart colors within node represent the proportion of individuals from each geographical location: Asia/Oceania = teal, CA-North = orange, CA-South = blue, Florida = pink, Hawaii = green, Mexico = yellow, Oregon = purple, Puerto Rico = brown, Texas = gray, and Unknown region from California = black.

Principal component analysis (PCA), discriminant analysis of principal components (DAPC), and minimum spanning network (MSN) analyses were conducted using a clone-corrected dataset, yielding results consistent with those obtained with non-clone-corrected data (**Figures 3**, **4**; **Supplementary Figure 2**). PCA did not reveal clustering by geographical locations; however, a clear separation between the two A2 panglobal clonal lineages was evident. Most Mexican and CA-South isolates grouped with the PcG2-A2 reference isolate, along with smaller subsets of isolates from CA-North, Hawaii and an unknown California location. In contrast, isolates from CA-North, CA-South, Florida, Hawaii, Texas, and Puerto Rico grouped with the PcG1-A2 reference isolate, together with small populations from Mexico and an unknown California location (**Figure 3A**). The Taiwanese population, comprising one A1 and one A2 isolate, grouped with the A1 isolate from CA-South and was clearly separated from the two A2 panglobal clonal lineages. Consistent with phylogenetic results, the three admixed isolates from CA-North and Hawaii were positioned between the A1 and A2 PNG reference isolates. DAPC provided further resolution of genetic relationships among populations. The analysis displayed clear separation between the Mexican and the other *P. cinnamomi* populations from this study. A small subset of isolates from CA-South, one isolate from CA-North, and one isolate from Hawaii were closely related with the Mexican population, as these isolates were all determined to be of the PcG2-A2 lineage (**Supplementary Figure 2B**). Consistent with the genetic differentiation results, isolates from Texas and Puerto Rico were more distantly related to the Mexican populations (**Table 1**; **Supplementary Figure 2B**).

Consistent with other analyses, the clone-corrected MSN showed that the U.S. and Mexican *P. cinnamomi* populations associated with avocado PRR, comprising 58 MLGs, belonged to the PcG1-A2 and PcG2-A2 panglobal lineages. These A2 clonal lineages were more distantly related to the A1 genotypes from PNG and CA-South, as well as to the Taiwanese A1 and A2 genotypes. The PcG1-A2 lineage comprised 30 nodes (MLGs), containing isolates from all sampled geographical locations. A large central node corresponding to MLG215 included two A2 reference isolates (PNG and Indonesia) and isolates from all U.S. regions and Mexico collected over multiple years, suggesting long-lived clonal lineages and little temporal differentiation. A smaller node (MLG137), containing isolates from CA-North, CA-South, and Hawaii, was also present within the PcG1-A2 lineage. The three unique MLGs corresponding to the admixed isolates from CA-North and Hawaii were also included in this lineage and were more closely related to CA-South genotypes. Evidence of additional clonal expansion was observed within this lineage across all U.S. regions, particularly in Puerto Rico (**Figure 4**; **Supplementary Figure 2C**). The PcG2-A2 clonal lineage (28 MLGs) comprised mainly genotypes from Mexico and CA-South. Interestingly, a small node (MLG 242) grouped genotypes from Mexico, CA-North, and Hawaii are also present in this lineage (**Figure 4**).

## 4 Discussion

In this study, we expanded previous *P. cinnamomi* population studies to include to all major avocado production regions across the United States, namely Florida, Hawaii, Texas, and Puerto Rico. By incorporating two reference isolates representing the two *P. cinnamomi* A2 panglobal clonal lineages reported by Shakya et al. (2021), we confirmed that the A2 clade I and A2 clade II previously reported in California and Mexico (Pagliaccia et al., 2013; Shands et al., 2025) correspond to the PcG1-A2 and PcG2-A2 panglobal clonal lineages, respectively.

The majority of *P. cinnamomi* isolates collected in this study from avocado-producing regions in California, Florida, Hawaii, Texas, and Puerto Rico clustered with the PcG1-A2 reference isolate (R024). These isolates also grouped with isolates from Mexico (PRS1_1 and TANR3_5), CA-South, CA-North, and several Asia/Oceania A2 reference isolates previously assigned to the A2 clade I by Shands et al. (2025), further confirming that this clade correspond to the PcG1-A2 panglobal lineage. In contrast, a subset of isolates from CA-South and one isolate from Hawaii clustered with the PcG2-A2 reference isolate (R001), indicating the presence of this panglobal lineage within *P. cinnamomi* populations associated with avocado PRR in the United States. These isolates clustered with the majority of the Mexican isolates, one isolate from CA-North (P344), and CA-South isolates previously described as A2 clade II isolates by Shands et al. (2025), allowing this clade to be assigned to the PcG2-A2 panglobal lineage and further confirming its presence in Mexico.

Shands et al. (2025) provided evidence supporting a Mexican origin of the A2 clade II, previously detected only in CA-South (Pagliaccia et al., 2013) now defined as PcG2-A2. Mexico is the world’s leading producer and exporter of ‘Hass’ avocado (FAO, 2025), and a long history of trade exists between Mexico and California, with increased avocado imports from Mexico over recent decades (USDA Foreign Agricultural Service, 2005). Movement of contaminated avocado fruits, seeds, and other plant material together with the movement of humans across the border of these two countries may explain the putative introduction of this lineage from Mexico to California. Notably, with the exception of one isolate from Hawaii, PcG2-A2 was not detected in other U.S. states included in this study. Florida and Puerto Rico primarily grow West Indian (WI) or WI hybrid cultivars grafted on WI rootstocks, as ‘Hass’ is not well adapted to local growing conditions (Crane et al., 2013). Consequently, movement of avocado scions and rootstocks between these states and Mexico is more limited than with California, which predominantly produces ‘Hass’, potentially explaining the absence of PcG2-A2 in these states. It is not clear why only PcG1-A2 clonal lineage was detected in Texas since there has been a history of germplasm imports to Texas historically from Mexico and from California and Florida since 1948 (Cooper, 1948; Nesbitt et al., 2013; Rouse and Knight, 1991). Apart from California, Hawaii which ranks third in avocado production in the United States (Hammami et al., 2024), was the only region where both panglobal lineages were detected, suggesting at least two independent introductions of *P. cinnamomi* into the state. *P. cinnamomi* has previously been recovered in Hawaii from avocado and other hosts including ornamentals (Kliejunas and Ko, 1976), thus, it is possible that these different lineages could have been introduced from contaminated avocado tissues or other hosts. It is possible that the relatively small sample sizes (12–25 isolates) per state limited the detection of PcG2-A2 in other regions. Additionally, including more reference isolates representing these panglobal lineages might have helped capture more genetic diversity within each lineage in relation to the populations assessed in this study.

The *P. cinnamomi* populations associated with avocado production in the United States exhibited a clonal mode of reproduction and low genetic differentiation, with pairwise *G_ST_* and *G′_ST_* values near zero. The greatest genetic differentiation was observed between Mexican and U.S. populations (**Table 1**). These findings suggest that *P. cinnamomi* introductions into U.S. avocado production may share common origins, potentially linked to historical germplasm exchanges or movement of contaminated plant material. Sexual recombination of *P. cinnamomi* in avocado orchards has not been reported in North America (Pagliaccia et al., 2013; Shands et al., 2025; Socorro Serrano et al., 2019) or South America (Calle-Henao et al., 2020), and we similarly found no evidence of sexual recombination within these populations. Shands et al. (2025) detected two isolates from CA-North (Pc2110 and P349) that were derived likely from a past sexual recombination event prior to its introduction. Similarly, in this study, we identified one admixed Hawaiian isolate (HI-5-SZ1) that shared ancestry with an A1 isolate from PNG (P3658, 60%) and the PcG1-A2 genetic cluster (40%), which includes the A2 PNG isolate (P6339) clustered with Pc2110 and P349 (**Figure 1**). Based on our phylogenetic and multivariate analyses, these three admixed isolates were more closely related to the PcG1-A2 than the PcG2-A2 isolates. Furthermore, A2 populations from this study were genetically related to reference isolates from PNG and showed evidence of ongoing clonal expansion within geographical regions (**Figures 3**, **4**; **Supplementary Figure 2**). Collectively, these results support the stepping-stone model of *P. cinnamomi* migration proposed by Shakya et al. (2021), in which South Africa, Australia, and Papua New Guinea acted as early recipients from Southeast Asia, followed by further clonal expansion to Europe, South America, and North America. To better assess these relationships and understand these migration patterns, more reference isolates from Asia and PNG regions would be added in future population studies.

Clone correction reduced the U.S. populations to 41 distinct MLGs, with each avocado-producing region having at least two MLGs that were unique. Both CA-South and CA-North populations were each represented by 15 MLGs. Previously, Shands et al. (2025) showed that the CA-South population was grouped in 27 MLGs when compared with 10 MLGs for the CA-North, suggesting more diversity for the CA-South population. The more balanced MLG distributions and similar pairwise *G_ST_* and *G′_ST_* values observed in this study suggest that the increased sampling size, expanded temporal range, and broader geographic coverage improved resolution of genetic relationships among populations. Consistent with this, the 50 Mexican isolates previously assigned to 50 unique MLGs (Shands et al., 2025) were resolved here into 13 unique, two shared, and four region-specific MLGs. With the exception of California, the *P. cinnamomi* populations from other U.S. regions are represented by < 6 MLGs suggesting low genetic differentiation in these regions and a larger genetic differentiation when compared with the population from Mexico and CA-South (**Table 1**). These results align with the fact that Mexico is the largest avocado producer in the world and California is the top producer in the United State while the avocado industry in the other regions is less than 10% (FAO, 2025; Hammami et al., 2024).

The high number of *P. cinnamomi* genotypes detected in Mexico and California may reflect increased local adaptation despite clonal reproduction, potentially driven by genome plasticity (Engelbrecht et al., 2021; Shands et al., 2024) and ploidy (Calle-Henao et al., 2020; Engelbrecht et al., 2021; Shands et al., 2024, Shands et al., 2025). Accordingly, broad phenotypic variability among isolates has been reported for attributes such as optimal growth temperature (Belisle et al., 2019a; Hoyt et al., 2026b; Shands et al., 2025), fungicide sensitivity (Belisle et al., 2019b; Hoyt et al., 2026b; Shands et al., 2025), and virulence (Belisle et al., 2019a; Hoyt et al., 2026b; Mondragón-Flores et al., 2022; Ochoa-Fuentes et al., 2015; Shands et al., 2025) in Mexico and California. Moreover, Hoyt et al. (2026b), phenotypically characterized the isolates from avocado used in this study collected from California (2020-2022) and other U.S. regions and found that these populations exhibited high adapted capacity to growing temperatures, fungicide, and virulence. All isolates analyzed here were estimated to be triploid (**Supplementary Table 2**). Oomycete pathogens are normally diploid, though different ploidy/aneuploidy levels have been reported within the Phytophthora genus including *P. cinnamomi* populations collected from avocado in Colombia (Calle-Henao et al., 2020), South Africa (Engelbrecht et al., 2021), California (Shands et al., 2024, Shands et al., 2025), and Mexico (Shands et al., 2025). Further analyses such as karyotyping and chromosome-level assemblies need to be conducted to confirm the ploidy of *P. cinnamomi* populations in this study.

Interestingly, we found several cases in which the single isolates from several unique MLGs were recently reported to exhibit high levels of potassium phosphite resistance (e.g., MLG156, EC_50_ up to 763.12 µg/ml) and adaptation to warmer temperatures (e.g., MLG45, optimal growth up to 28 °C) (Hoyt et al., 2026b). Additionally, Pc2109 and Pc2117, previously reported as highly virulent isolates (Belisle et al., 2019a; Shands et al., 2025; Hoyt et al., 2026b) were both assigned to MLG21. Further studies are required to determine the correlation between phenotypes and MLG assignments. On the other hand, MLG215, comprising the largest number of isolates, spanned all avocado-growing regions, and included two A2 reference isolates from PNG and Indonesia (**Figure 4**). Isolates within MLG215 exhibited wide phenotypic variation both within and among regions, including potassium phosphite EC_50_ values ranging from 7.2 to 580.6 µg/ml and virulence levels ranging from 29% to 80% diseased roots. It is intriguing to find such extreme phenotypic variation within closely related genotypes. Research is undergoing to determine the molecular basis of these phenotypic variability. Our minimum spanning network analyses suggest that MLG215 may function as a hub for clonal expansion and dissemination of these adaptive genotypes across avocado-producing regions, posing challenges to current PRR management strategies and threatening industry sustainability.

In conclusion, we expanded the characterization of *P. cinnamomi* population structure and genetic differentiation from California and Mexico to other avocado-growing regions in the United States. Our results reclassified the two California A2 clonal lineages (Pagliaccia et al., 2013; Shands et al., 2025) as the two panglobal A2 clonal lineages described by Shakya et al. (2021). PcG1-A2 was detected across all regions surveyed, whereas PcG2-A2 was limited to California, Mexico, and Hawaii. The clonal pathogen populations were partitioned into 61 region-specific, shared, and unique MLGs, with greater differentiation observed between U.S. and Mexican populations than among U.S. regions, reflecting the dominant presence of PcG2-A2 in Mexico and PcG1-A2 in the U.S. By integrating genomic and phenotypic data, this study provides a robust foundation for future research aimed at elucidating the molecular mechanisms underlying genotypic and phenotypic diversity within clonal lineages. Finally, the genomic resources generated here will support the development of effective and durable PRR management strategies, including lineage-, isolate-, and trait-specific diagnostic tools to monitor the emergence and spread of *P. cinnamomi* adapted to current control methods across the United States and Mexico.

## Data Availability

Whole-genome sequences have been deposited to the Sequence Read Archive under NCBI BioProject accession PRJNA1459401.

## Supplementary material

Supplementary Table 1 *Phytophthora cinnamomi* isolates used in this study.

Supplementary Table 2 nQuire ploidy estimation results of denoised bin files. The free, dip (diploid), tri (triploid) and tet (tetraploid) columns represent the respective fixed model maximized log-likelihood, and the ∆ dip, ∆ tri, and ∆ tet represent the respective delta log-likelihood. The fixed model values closest to the free (bold) and the delta loglikelihood with the lowest value (bold) supports the respective ploidy level.

Supplementary Table 3 Clone correction of the *P. cinnamomi* populations. *Phytophthora cinnamomi* isolates were assigned to their respective multi-locus genotype (MLG) groups after applying an MLG filter of 0.01 using *poppr.*

Supplementary Figure 1 FastSTRUCTURE distruct plots displaying the posterior probability assignment of genetic clusters using the most likely K values selected by fastSTRUCTURE using the logistic prior (K = 3, 4, and 5). Each bar represents an individual and the bar color represents the likelihood of the individual to be assigned to a genetic cluster. Geographic locations are displayed beneath these plots.

Supplementary Figure 2 Multivariate analyses of the *P. cinnamomi* populations from the U.S.A. and Mexico using non-clone corrected data. The points in each plot represent the individual by geographical location: Asia/Oceania = teal, CA-North = orange, CA-South = blue, Florida = pink, Hawaii = green, Mexico = yellow, Oregon = purple, Puerto Rico = brown, Texas = gray, and Unknown region from California = black. **(A)** Principal component analysis (PCA) plot showing two principal components in which PC1 reflects 84.37% and PC2 reflects 3.96% of the total variation. **(B)** Discriminant analysis of principal components (DAPC) plot indicating the different PC groups where the points represent the individuals, and the ellipses represent the geographical locations. DA eigenvalues distribution is also displayed. (**C**) Minimum spanning network (MSN) generated using pairwise distances between each isolate where edge thickness represents the genetic distance. *Phytophthora cinnamomi* reference isolates corresponding to the two A2 panglobal clonal lineages (PcG1-A2 and PcG2-A2), several A1 and A2 isolates from Taiwan, Papua Guinea (PNG), and Indonesia, as well an A1 isolate from CA-South collected from Camelia are indicated in **(A, B)**.
